# Programmed-Cell-Death-Related Signature Reveals Immune Microenvironment Characteristics and Predicts Therapeutic Response in Diffuse Large B Cell Lymphoma

**DOI:** 10.3390/biomedicines13102320

**Published:** 2025-09-23

**Authors:** Donghui Xing, Kaiping Luo, Xiang He, Xin Hu, Yixin Zhai, Yanan Jiang, Wenqi Wu, Zhigang Zhao

**Affiliations:** 1 Department of Medical Oncology, Tianjin First Central Hospital, School of Medicine, Nankai University, Tianjin 300192, China; 15350735509@163.com (D.X.); luokaiping2022@163.com (K.L.); zzhai2020@tmu.edu.cn (Y.Z.); yananjiang@tmu.edu.cn (Y.J.); 2 Department of Oncology, Tianjin Medical University Cancer Institute and Hospital, Tianjin 300060, China; 15733992690@163.com; 3 Tianjin Medical University Cancer Institute and Hospital, National Clinical Research Center for Cancer, Tianjin 300060, China; tmu2018huxin@163.com (X.H.); wenqiwu1@163.com (W.W.); 4 Key Laboratory of Cancer Prevention and Therapy, Tianjin 300060, China; 5 Tianjin’s Clinical Research Center for Cancer, Tianjin 300060, China; 6 Department of Epidemiology and Biostatistics, Tianjin Medical University Cancer Institute and Hospital, Tianjin 300060, China; 7 Key Laboratory of Molecular Cancer Epidemiology, Tianjin 300060, China; 8 Department of Laboratory Medicine, Zhongnan Hospital of Wuhan University, Wuhan 430071, China; 9 Department of Senior Ward, Tianjin Medical University Cancer Institute and Hospital, Tianjin 300060, China

**Keywords:** DLBCL, prognosis, chemotherapy, TME, programmed cell death patterns

## Abstract

**Background/Objectives**: Diffuse large B cell lymphoma (DLBCL) is a highly heterogeneous and aggressive lymphoma with a high incidence rate. Although modern therapeutic approaches have significantly improved patient survival rates, treatment relapse and drug resistance remain major clinical challenges. Programmed cell death (PCD) promotes tumorigenesis and regulates the tumor microenvironment (TME) and drug sensitivity. Exploring the application potential of PCD in DLBCL could pave the way for new treatment strategies for this malignancy. **Methods**: We systematically analyzed 13 types of PCD pathways and integrated transcriptomic and clinical data from 832 DLBCL patients (GSE10846, GSE11318, and GSE87371). A PCD-based prognostic signature, termed the Programmed Cell Death Score (PCDS), was constructed using 20 key PCD-related genes. Its clinical relevance was evaluated through survival analysis, drug response profiling, and tumor immune infiltration assessment using CIBERSORT, ESTIMATE, and ssGSEA algorithms. **Results**: The PCDS robustly stratified patients by survival and outperformed conventional clinical indicators such as age, stage, Eastern Cooperative Oncology Group (ECOG), and lactate dehydrogenase (LDH) in prognostic prediction. High-PCDS tumors were associated with immune suppression, characterized by reduced CD8^+^ T cell infiltration, elevated M2 macrophages, and increased programmed cell death protein 1 (PD-1)/programmed cell death ligand 1 (PD-L1) expression. Drug sensitivity analysis revealed that high-PCDS patients may benefit more from agents like sorafenib and fulvestrant, while low-PCDS patients responded better to NU7441. Functional validation using DLBCL cell lines and xenografts confirmed the oncogenic role of a representative gene (*CTH*) within the model. **Conclusions**: This study presents a novel prognostic scoring system derived from multiple PCD pathways that effectively stratifies DLBCL patients by risk and therapeutic responsiveness. Notably, the PCDS is closely associated with key immunological characteristics of the TME. These findings advance personalized treatment strategies and support clinically relevant decision-making in DLBCL.

## 1. Introduction

Diffuse large B cell lymphoma (DLBCL) is a malignant hematopoietic disease characterized by rapid clinical progression and high incidence rates [1,2]. The introduction of rituximab and immunosuppressants has substantially improved patient outcomes. However, 30% to 40% of patients still experience relapse or face challenges with treatment resistance [1,2]. Enhancing prognostic stratification and developing precise treatment strategies are therefore crucial for further improving patient prognosis. Conventional clinical tools such as the International Prognostic Index (IPI) have long been used to estimate outcomes in DLBCL. However, the IPI relies on generalized clinical parameters, such as age, stage, and lactate dehydrogenase (LDH), and fails to capture the molecular complexity of the disease. With the advent of high-throughput sequencing and large-scale omics technologies, the molecular taxonomy of DLBCL has undergone considerable refinement. Studies have identified at least two primary molecular subtypes—germinal center B cell-like (GCB) and activated B cell-like (ABC)—with distinct gene expression profiles and prognostic implications. These findings have highlighted the necessity of integrating molecular data into prognostic modeling and treatment decision-making.

Programmed cell death (PCD) is a tightly regulated biological process that not only maintains tissue homeostasis but also modulates anti-tumor immunity. While apoptosis has long been recognized as the canonical form of PCD, recent studies have identified a diverse array of alternative PCD modalities, including disulfidptosis, pyroptosis, ferroptosis, cuproptosis, necroptosis, parthanatos, autophagy-dependent cell death, alkaliptosis, netotic cell death, entotic cell death, oxeiptosis, and lysosome-dependent cell death [2,3,4,5,6]. Each of these forms represents a unique regulatory mechanism with critical implications for cancer progression and therapeutic resistance.

These non-apoptotic forms of PCD are increasingly appreciated not only for their roles in tumor progression and resistance but also for their profound impact on the tumor immune microenvironment. Several studies have demonstrated that activating these non-apoptotic PCD pathways can sensitize tumors to chemotherapy or immunotherapy. For example, ferroptosis is linked to lipid peroxidation and enhanced sensitivity to immune checkpoint blockade [7,8]. Pyroptosis can elicit robust anti-tumor immunity by releasing damage-associated molecular patterns (DAMPs), thereby attracting immune cells [9,10,11]. Cuproptosis, a copper-dependent modality that targets lipoylated mitochondrial enzymes, has opened a new avenue for metabolic vulnerabilities in cancer [12]. More recently, disulfidptosis was identified as cytoskeletal collapse induced by disulfide stress, offering novel insights into redox regulation of cancer cell survival [4]. These discoveries, consolidated in recent comprehensive reviews [8,9,13], highlight the therapeutic potential of modulating PCD pathways to enhance cancer immunotherapy. However, despite these advances, most mechanistic and translational studies have been confined to solid tumors, whereas the clinical and prognostic relevance of non-apoptotic PCD pathways in hematological malignancies remains poorly understood. DLBCL, the most common and heterogeneous subtype of non-Hodgkin lymphoma, exemplifies this knowledge gap. Conventional prognostic tools cannot fully capture the molecular complexity and immune heterogeneity of the disease. Given the increasing role of immunotherapy in DLBCL, there is an urgent need to investigate how diverse PCD pathways intersect with the tumor microenvironment (TME) to influence patient outcomes. Addressing this gap may provide novel biomarkers and therapeutic strategies tailored to the unique biology of DLBCL.

In this study, we systematically characterized 13 PCD pathways in DLBCL using transcriptomic data from large public cohorts. We developed a novel Programmed Cell Death Score (PCDS) that integrates gene signatures across multiple PCD types to predict patient prognosis. Beyond survival prediction, we further evaluated differences in drug sensitivity and tumor immune microenvironment characteristics across PCDS-defined subgroups. Experimental validation of a key model gene (*CTH*) in both in vitro and in vivo systems confirmed its functional role in tumor progression. In conclusion, our findings provide a biologically informed and clinically applicable model that connects cell death regulation to tumor–immune interactions. The PCDS may serve as a novel stratification tool and a potential guide for personalized treatment strategies in DLBCL, especially in the context of immunotherapy and tumor microenvironment-targeted approaches.

## 2. Materials and Methods

### 2.1. Data Collection

PCD genes were curated from multiple authoritative sources, including the FerrDb V2 database, MSigDB gene sets from the Gene Set Enrichment Analysis (GSEA) resource, and recent high-impact review articles, to ensure comprehensive coverage of diverse PCD types (Table S1). Transcriptomic profiles and corresponding clinical data for DLBCL samples were extracted from three GEO datasets: GSE10846, GSE11318, and GSE87371. After removing duplicate entries and samples lacking complete clinical annotations, a total of 832 high-quality DLBCL patient samples were included for downstream analysis. Data were normalized and batch-corrected prior to integration.

### 2.2. A Prognosis Model Development and Validation

In the training cohort GSE10846, we screened 481 prognostic genes with *p* < 0.05 by univariate Cox survival analysis (Table S2). Twenty core genes were screened with the optimal lambda value (“lambda.min”) as the threshold. The following risk score was also constructed: PCDS = $\sum_{i=1}^{20}Coef\;*\;geneexpression$. The model’s validity was further confirmed in the GSE11318 and GSE87371 datasets using Kaplan–Meier survival curves. Additionally, the independence of the PCDS was verified through multivariate Cox regression analyses.

### 2.3. Gene Set Enrichment Analysis

Differential gene expression analysis was performed using the “limma” R package (version 3.61.5), followed by gene set enrichment analysis using the “clusterProfiler” R package (version 4.14.6).

### 2.4. Nomogram Construction and Validation

We constructed a nomogram integrating the PCDS with established clinical variables, including age and clinical stage, using the “regplot” package (version 1.1) in R. Calibration curves were plotted using the “rms” package (version 8.0.0) to assess the predictive performance and accuracy of the nomogram.

### 2.5. Drug Sensitivity and TME Analysis

We utilized the Gene Set Cancer Analysis (GSCA) platform and the “oncoPredict” R package to predict chemotherapeutic sensitivity based on transcriptomic profiles. Drug response was evaluated across PCDS-defined subgroups to identify differential sensitivities. For TME analysis, we employed three complementary algorithms. ESTIMATE was used to calculate immune and stromal scores, providing an overall assessment of immune and stromal cell infiltration. CIBERSORT was applied to deconvolute bulk transcriptomic data into 22 immune cell subsets. Only samples with a CIBERSORT output *p* < 0.05 were retained for downstream analysis. ssGSEA was conducted to quantify enrichment scores of predefined immune-related gene sets, including T cell activation and cytokine signaling. These analyses provided a comprehensive view of immune infiltration and functionality in high- and low-PCDS groups.

### 2.6. Cell Culture

The DLBCL cell lines DOHH2 and OCI-LY7 were purchased from the American Type Culture Collection (ATCC, Manassas, VA, USA). Cell line authentication was performed by short tandem repeat (STR) profiling, and all cell lines were routinely tested to ensure they were free of mycoplasma contamination. Cells were maintained in RPMI-1640 medium (Gibco, Thermo Fisher Scientific, Carlsbad, CA, USA) supplemented with 10% fetal bovine serum (FBS; Gibco, Thermo Fisher Scientific, Carlsbad, CA, USA) and 1% penicillin–streptomycin solution at 37 °C in a humidified incubator containing 5% CO_2_. Cells in the logarithmic growth phase were used for subsequent experiments.

### 2.7. Knockdown of the Key Model Gene CTH

GeneChem (Shanghai, China) designed and synthesized specific shRNAs targeting human *CTH* and subcloned them into the GV112 lentiviral expression vector (hU6-MCS-CMV-Puromycin). Lentiviruses were packaged and used to transduce DLBCL cell lines (DOHH2 and OCI-LY7), followed by puromycin selection. Cellular proliferation was assessed using 5-ethynyl-2′-deoxyuridine (EdU) incorporation assays, while apoptosis was quantified using Annexin V conjugated with fluorescein isothiocyanate (Annexin V–FITC) and propidium iodide (PI) dual staining, followed by flow cytometric analysis to distinguish and measure apoptotic cell populations.

### 2.8. Tumor Xenograft

Animal experiments were performed in accordance with the regulations of the Animal Ethics and Welfare Committee of Tianjin Medical University. OCI-LY7 cells transduced with either *CTH*-targeting shRNA (sh*CTH*) or a control shRNA (shNT) were harvested, washed, and resuspended in PBS/Matrigel (1:1) at a density of 5 × 10^6^ cells/100 µL. The cell suspension was injected subcutaneously into the flanks of 6–8-week-old female NOD/SCID mice (n = 5 per group). Tumor growth was monitored every 2–3 days, and tumor volume was calculated using the following formula: volume (mm^3^) = (length × width^2^) × 0.5.

### 2.9. Statistical Analysis

All statistical analyses in this paper were performed using R.4.4.1 and GraphPad software (version 8.0.2). Kaplan–Meier curves assessed survival, and multivariate Cox regression determined the independence of the PCDS. The Wilcoxon rank sum test was used for continuous variables with non-normal distribution. *p* < 0.05 was considered statistically significant, and both tests were bilateral.

## 3. Results

### 3.1. Construction of a Prognostic Model for DLBCL Patients

**Figure 1:**
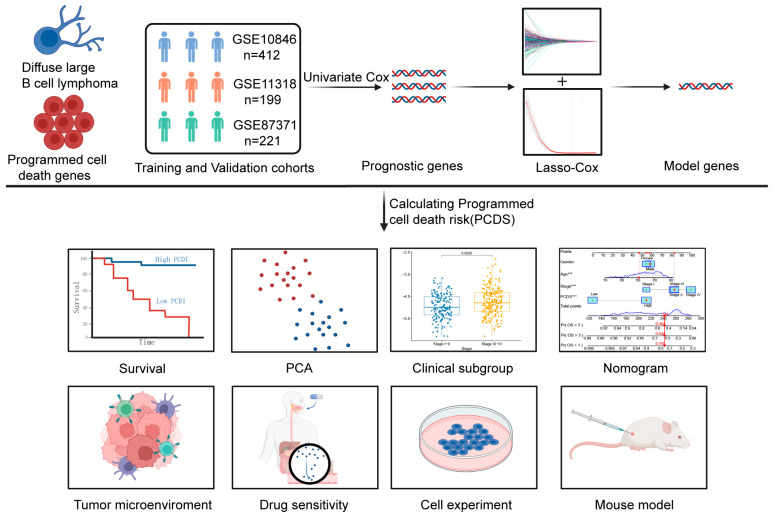
A comprehensive flowchart of this study.

The detailed workflow for constructing and validating a PCDS-based prognostic model is presented in Figure 1. We first curated 1382 genes associated with 13 distinct forms of PCD from FerrDb V2, MSigDB, and sources from the literature (Table S1). Using univariate Cox proportional hazards regression analysis in the GSE10846 dataset (n = 412), we identified 481 genes significantly correlated with overall survival (OS) (*p* < 0.05) (Table S2).

**Figure 2:**
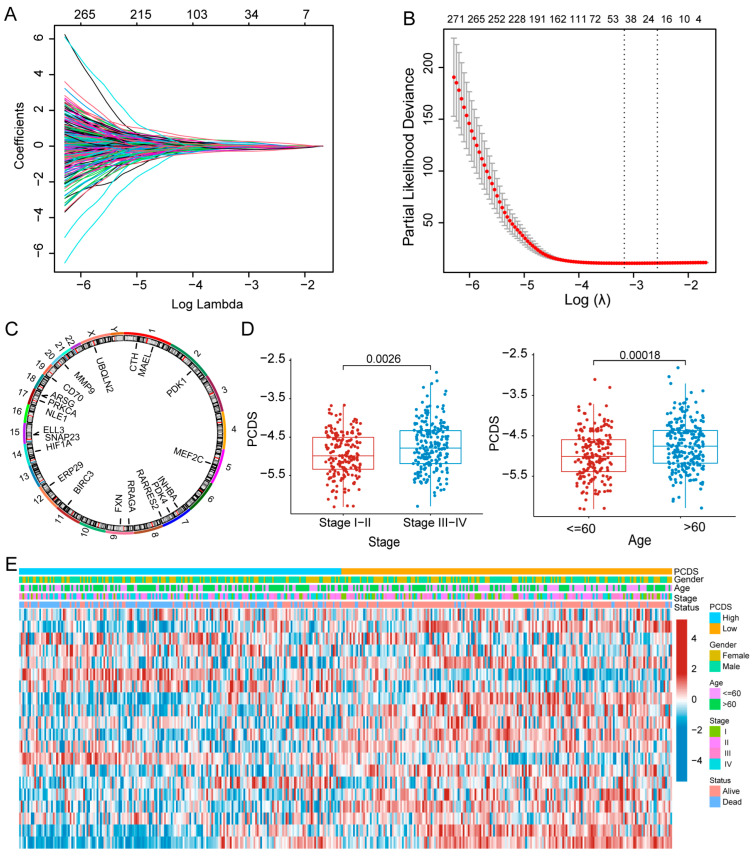
Identification of core model genes and clinical correlations with the PCDS in DLBCL patients. (**A**,**B**) LASSO regression analysis of 481 prognosis-associated genes from the GSE10846 dataset (n = 412). The cross-validation curve and lambda curves are shown, with the optimal λ value selected by the minimum partial likelihood deviance. (**C**) Genomic chromosomal distribution of the 20 genes incorporated into the PCDS model. (**D**) Distribution of PCDS values across clinical subgroups stratified by stage (I–II vs. III–IV) and age (<60 vs. ≥60 years). (**E**) Heatmap illustrating the association between the expression levels of the 20 model genes and major clinical features. Abbreviations: PCDS, programmed cell death score.

To avoid overfitting and reduce dimensionality, LASSO–Cox regression was performed, with the optimal λ selected based on the lowest partial likelihood deviance (Figure 2A,B). This resulted in the selection of 20 core prognostic genes: *ARSG*, *BIRC3*, *CD70*, *CTH*, *ELL3*, *ERP29*, *FXN*, *HIF1A*, *INHBA*, *MAEL*, *MEF2C*, *MMP9*, *NLE1*, *PDK1*, *PDK4*, *PRKCA*, *RARRES2*, *RRAGA*, *SNAP23*, and *UBQLN2.* The chromosomal locations of these genes are presented in Figure 2C. These genes are associated with critical PCD pathways, including apoptosis, ferroptosis, autophagy, lysosome-dependent cell death, and necroptosis. We used these genes to develop a risk scoring tool called the PCDS and divided patients into two subgroups of high and low PCDSs based on the median PCDS. Clinical subgroup analyses revealed that PCDS values were significantly correlated with age and clinical stage (I–IV). Specifically, patients with Stage III–IV disease or older age (≥60 years) were more likely to exhibit higher PCDS values (Figure 2D,E and Figure S1A,B).

To explore the molecular mechanisms differentiating PCDS subgroups, we conducted a GSEA. In the high-PCDS subgroup, enrichment was observed in metabolic and DNA damage repair pathways, including galactose metabolism, homologous recombination, and nitrogen metabolism, suggesting a metabolic reprogramming and increased genomic instability in this group (Figure S2A). Conversely, the low-PCDS subgroup showed enrichment in immune regulatory pathways such as adherens junction, gap junction, and TGF-beta signaling, implying enhanced immune interactions and epithelial features (Figure S2B). Collectively, these findings indicate that the PCDS is not only a statistically robust prognostic marker but also reflects underlying biological heterogeneity.

### 3.2. Validation of the PCDS-Based Prognostic Model

**Figure 3:**
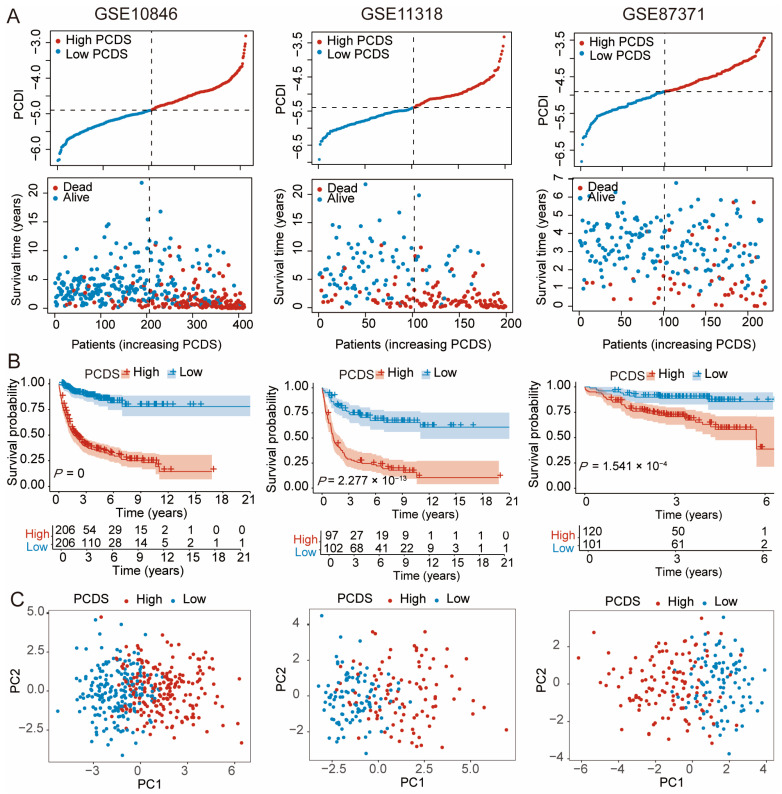
Validation of the prediction model using training and external datasets. (**A**) PCDS distribution over time and survival status in three independent GEO cohorts (GSE10846, n = 412; GSE11318, n = 199; GSE87371, n = 221). (**B**) Kaplan–Meier overall survival curves comparing high- and low-PCDS groups in the three cohorts. Survival differences were evaluated using the log-rank test. (**C**) PCA plots showing distinct separation of patients into high- and low-PCDS groups according to the expression profiles of the 20 model genes. Abbreviations: PCDS, programmed cell death score.

To assess the robustness and universality of the PCDS model, we validated its prognostic performance across three independent GEO cohorts: GSE10846, GSE11318, and GSE87371. Patients in each dataset were divided into high- and low-PCDS groups according to the median score. The distribution of PCDS values and corresponding survival status clearly showed that higher PCDS was associated with increased mortality across all cohorts (Figure 3A). Kaplan–Meier survival curves revealed that patients with higher PCDS values exhibited worse survival outcomes (Figure 3B; GSE10846: *p* = 0, GSE11318: *p* = 2.27 × 10^−13^, GSE87371: *p* = 5.41 × 10^−4^), confirming the prognostic value of the PCDS. To evaluate the classification ability of the model, principal component analysis (PCA) was conducted using the expression profiles of the 20 model genes. The PCA plots demonstrated distinct separation between high- and low-PCDS subgroups in all three cohorts (Figure 3C).

Further subgroup analyses based on clinical parameters revealed that the PCDS retained strong prognostic significance across diverse clinical settings. As shown in Figure S3A–C, both younger (≤60 years) and older (>60 years) patients exhibited significant differences in survival between high- and low-PCDS groups in the GSE10846 and GSE11318 cohorts. Moreover, the PCDS remained a robust predictor in both Stage I–II and Stage III–IV patients. Even in the smaller GSE87371 cohort, the model maintained moderate prognostic separation, particularly in patients with Stage III–IV disease (*p* = 0.010).

These results underscore the stability and applicability of the PCDS across independent datasets and various clinical subgroups. Collectively, the consistent performance of the PCDS in stratifying patient risk affirms its clinical utility as a reliable prognostic biomarker in DLBCL.

### 3.3. Development and Evaluation of the Nomogram-Based Survival Model

**Figure 4:**
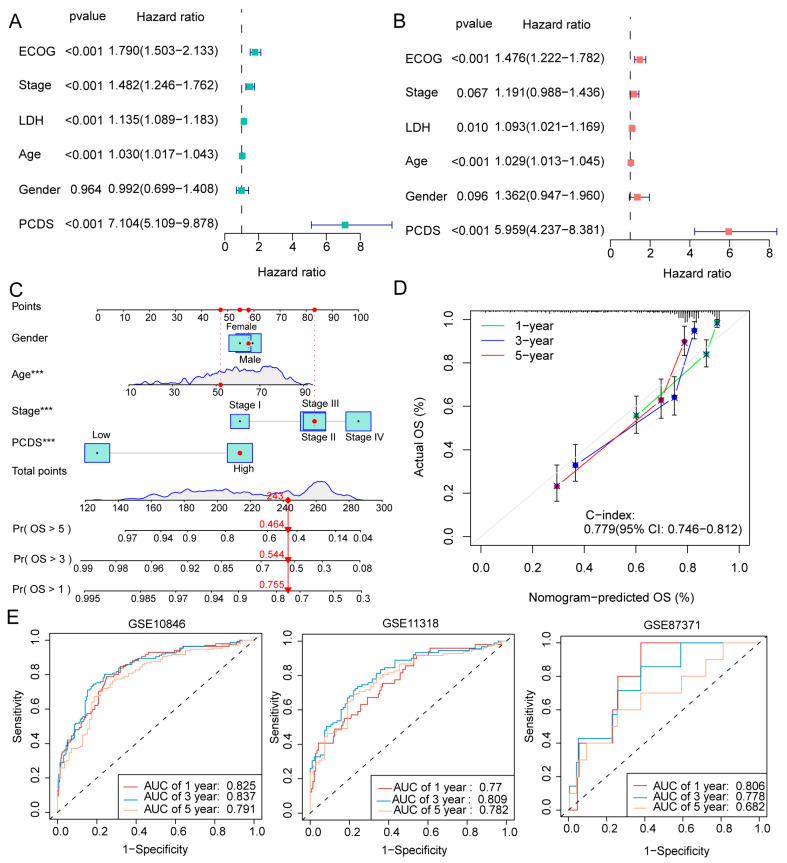
Development and evaluation of the nomogram-based survival model. (**A**,**B**) Univariate and multivariate Cox regression analyses of OS, including PCDS and established clinical variables (age, stage, LDH, ECOG, gender). Hazard ratios (HRs) with 95% CIs are displayed. (**C**) Nomogram constructed from PCDS and clinical parameters to predict 1-, 3-, and 5-year OS in DLBCL patients. (**D**) Calibration plots demonstrating the concordance between predicted and observed OS at 1, 3, and 5 years. (**E**) Receiver operating characteristic (ROC) curves showing the predictive performance of the nomogram in three independent cohorts (GSE10846, GSE11318, GSE87371). AUC values are reported for each time point. *** *p* < 0.001. Abbreviations: AUC, area under the curve; CI, confidence interval; C-index, concordance index; ECOG, Eastern Cooperative Oncology Group; HR, hazard ratio; LDH, lactate dehydrogenase; OS, overall survival; PCDS, programmed cell death score.

Univariate Cox regression analysis was performed to identify the significant predictors of OS in DLBCL patients. The analysis revealed that the PCDS, age, stage, LDH levels, and Eastern Cooperative Oncology Group (ECOG) performance status were significant prognostic factors for OS (Figure 4A). This was further corroborated by multivariate analysis, where the PCDS maintained its role as an independent predictor of OS after adjusting for confounders (Figure 4B), supporting its robustness across clinical parameters.

Building upon these findings, we constructed a nomogram incorporating key variables, including gender, age, clinical stage, and PCDS, to enhance the accuracy of prognostic prediction (Figure 4C). The nomogram aims to provide a more personalized risk prediction for patients, with an easy-to-use graphical tool that clinicians can apply in clinical practice. Calibration curves were used to assess the predictive performance of the nomogram; the curves showed that the predicted 1-, 3-, and 5-year OS rates closely matched the observed survival rates, highlighting the model’s strong predictive power (Figure 4D). The model’s accuracy was further validated with a concordance index (C-index) of 0.779, which fell within the 95% confidence interval (CI) of 0.746–0.812, indicating good consistency between the predicted and actual OS outcomes.

Additionally, the nomogram was validated using three independent cohorts (GSE10846, GSE11318, and GSE87371). The area under the curve (AUC) values for 1-year, 3-year, and 5-year OS predictions were calculated and demonstrated good discrimination ability, with AUC values of 0.825, 0.837, and 0.791 for GSE10846; 0.77, 0.809, and 0.782 for GSE11318; and 0.806, 0.778, and 0.682 for GSE87371 (Figure 4E). These findings collectively underscore the robustness of the PCDS-based nomogram in predicting clinical outcomes and demonstrate its potential to guide personalized therapeutic strategies in DLBCL, highlighting the prognostic utility of the PCDS-based risk model for clinical outcomes in DLBCL.

### 3.4. TME Dissection Based on ESTIMATE, ssGSEA, and CIBERSORT Analyses

**Figure 5:**
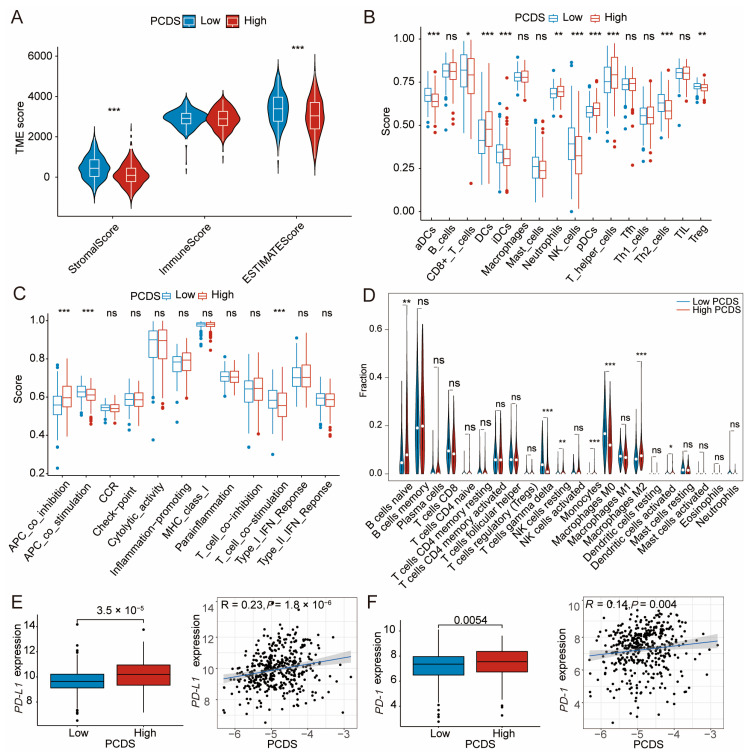
Tumor immune microenvironment analysis based on PCDS subgroups. (**A**) ESTIMATE algorithm-derived stromal and immune scores in high- vs. low-PCDS groups. (**B**) ssGSEA ImmCell infiltration analysis. (**C**) ssGSEA ImmFunction analysis. (**D**) CIBERSORT immune cell composition analysis. (**E**,**F**) Boxplots and correlation plots illustrating the positive association between PCDS values and the expression of immune checkpoint molecules PD-1 and PD-L1. ns, not significant; * *p* < 0.05; ** *p* < 0.01; *** *p* < 0.001. Abbreviations: PCDS, programmed cell death score; TME, tumor microenvironment; aDC, activated dendritic cell; APC, antigen-presenting cell; CCR, cytokine–cytokine receptor interaction; IFN, interferon; PD-1, programmed cell death protein 1; PD-L1, programmed cell death ligand 1.

The TME significantly influences tumor progression and therapeutic outcomes. We used a variety of immune algorithms to perform immune analysis. The ESTIMATE algorithm revealed that StromalScore was significantly lower in patients with a high PCDS (Figure 5A). Consistently with this, ssGSEA ImmCell analysis showed a notable reduction in the infiltration of critical immune cells, such as CD8+ T cells and activated dendritic cells (aDCs), in patients with a high PCDS, reflecting diminished immune activity within the tumor microenvironment (Figure 5B) [14,15,16,17]. ssGSEA ImmFunction further highlighted significant impairments in critical immune pathways in the high-PCDS group, such as antigen-presenting cell (APC) co-stimulation and T cell activation (Figure 5C). Moreover, CIBERSORT analysis confirmed reduced proportions of anti-tumor immune cells, such as M1 macrophages in patients with a high PCDS. Conversely, there was a notable increase in M2 macrophages associated with immunosuppression (Figure 5D). Additionally, we observed higher infiltration levels of M0 macrophages and T cells gamma delta in the low-PCDS group. Survival analysis showed that high infiltration of M0 macrophages and T cells gamma delta was strongly associated with improved survival outcomes (Figure S4A,B). Conversely, M2 macrophage infiltration increased in the high-PCDS group, and the prognosis was poor (Figure S4C). These findings further emphasized the impact of immune cell infiltration on patient outcomes. We also examined immune checkpoint molecule expression, revealing that the high-PCDS group exhibited markedly elevated programmed cell death protein 1 (PD-1) and programmed cell death ligand 1 (PD-L1) levels (Figure 5E,F). A positive correlation was observed between PCDS values and PD-1 and PD-L1 expression, further confirming the immunosuppressive nature of tumor microenvironment in the high PCDS group. In summary, reactivating anti-tumor immune responses is essential to improving clinical outcomes in this subgroup of high-PCDS patients.

### 3.5. Heterogeneity of Drug Sensitivity Based on PCDS Values

**Figure 6:**
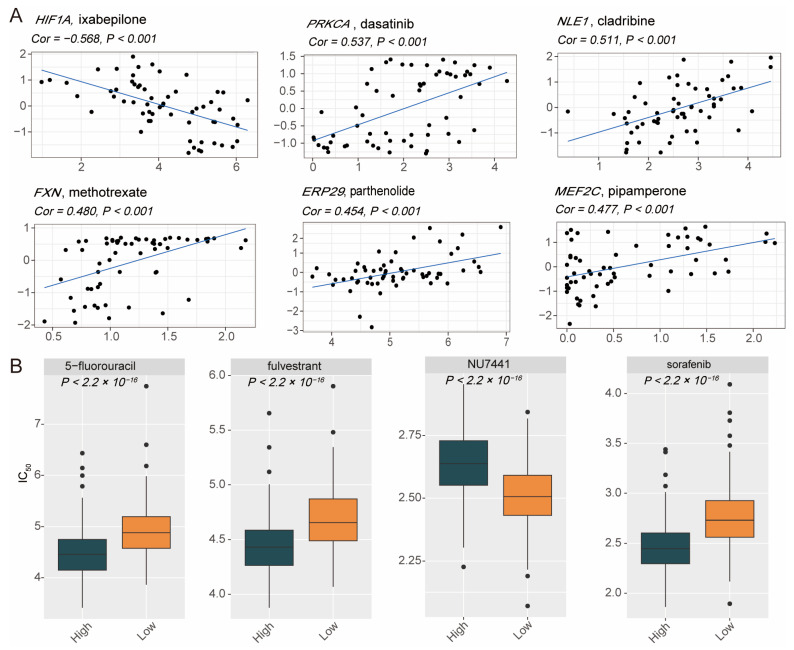
Correlations between drug sensitivity and PCDS. (**A**) Correlation matrix between the expression of individual PCDS model genes and predicted sensitivity to chemotherapeutic or targeted drugs. Drug sensitivity was assessed based on IC_50_ values estimated from the GDSC database using ridge regression models in the “oncoPredict” R package (v0.3.0). (**B**) Predicted drug sensitivity differences between high- and low-PCDS groups, showing significantly lower IC_50_ values for 5-fluorouracil, fulvestrant, and sorafenib in the high-PCDS subgroup and increased sensitivity to NU7441 in the low-PCDS subgroup. Comparisons were performed using the Wilcoxon rank sum test, with *p* < 0.05 considered statistically significant. Abbreviations: IC_50_, half maximal inhibitory concentration.

We analyzed the differences in treatment response between the two groups of DLBCL patients to optimize precision treatment and prognosis. First, correlation analyses were conducted between model gene expression and drug response (Figure 6A). The results revealed that higher expression levels of *PRKCA*, *NLE1*, *FXN*, *ERP29*, and *MEF2C* were positively correlated with increased sensitivity to specific drugs, such as dasatinib, cladribine, methotrexate, parthenolide, and pipamperone, respectively. Conversely, *HIF1A* expression was negatively correlated with sensitivity to ixabepilone, indicating its potential role in mediating resistance to certain therapies. Additionally, we further investigated drug response difference between high- and low-PCDS groups (Figure 6B). The high-PCDS group exhibited significantly lower half maximal inhibitory concentration (IC_50_) values for 5-fluorouracil, fulvestrant, and sorafenib, indicating greater sensitivity to these treatments. On the other hand, the low-PCDS group demonstrated greater sensitivity to NU7441, with significantly lower IC_50_ values compared to the high-PCDS group. These findings highlight the heterogeneity in drug sensitivity among DLBCL patients stratified by PCDS values. The high-PCDS group appears to benefit more from specific targeted therapies, whereas the low-PCDS group may respond better to other agents like NU7441. This underscores the importance of PCDS-based stratification in guiding personalized therapeutic strategies for DLBCL patients.

### 3.6. Functional Effects of CTH Knockdown on DLBCL Cells

**Figure 7:**
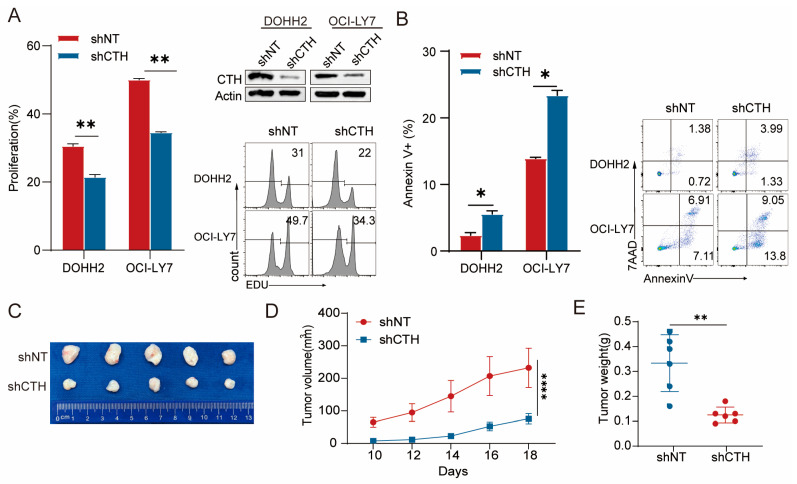
Functional role of *CTH* knockdown in DLBCL. (**A**) Western blot and EdU incorporation assays showing that *CTH* knockdown significantly reduced cell proliferation in DOHH2 and OCI-LY7 cell lines compared to shNT. (**B**) Flow cytometry analysis of Annexin V/PI staining demonstrating that *CTH* silencing induced apoptosis in DOHH2 and OCI-LY7 cells. (**C**) Representative images of xenograft tumors generated by OCI-LY7 cells transduced with sh*CTH* or shNT. (**D**,**E**) Tumor volume and endpoint tumor weights showing significant suppression of in vivo tumor growth following *CTH* knockdown (n = 5 mice per group). Data are presented as mean ± SEM, and differences were analyzed by Student’s t test; *p* < 0.05 was considered statistically significant. * *p* < 0.05; ** *p* < 0.01; **** *p* < 0.0001.

Currently, the impact of *CTH* in DLBCL remains unexplored. To bridge this gap, we conducted functional studies to evaluate its significance. First, Western blotting confirmed effective knockdown of *CTH* expression in DOHH2 and OCI-LY7 cell lines using sh*CTH* compared to shNT (Figure 7A). EdU incorporation assays demonstrated that *CTH* knockdown significantly suppressed cell proliferation in both cell lines, indicating a key role of *CTH* in promoting DLBCL cell growth. In parallel, flow cytometry analysis of Annexin V/7-AAD-stained cells revealed markedly elevated levels of apoptosis in *CTH*-silenced cells, further supporting its pro-survival function (Figure 7B). To evaluate the in vivo relevance of *CTH*, we established a xenograft tumor model by subcutaneously injecting OCI-LY7 cells transduced with either sh*CTH* or shNT into immunodeficient mice. Tumor growth was monitored over time, and the results showed that tumors formed by sh*CTH* cells exhibited significantly reduced growth rates and final volumes compared to the control group (Figure 7C,D). Moreover, tumor weights at endpoint were significantly lower in the sh*CTH* group, confirming the tumor-suppressive effect of *CTH* knockdown (Figure 7E). Together, these findings indicate that *CTH* acts as a functional driver in DLBCL by promoting tumor cell proliferation and inhibiting apoptosis and may be a potential target for DLBCL therapy.

## 4. Discussion

DLBCL is a highly heterogeneous malignancy with diverse molecular subtypes, clinical characteristics, and responses to treatment. This diversity poses significant challenges to effective risk stratification and individualized treatment. Although therapeutic advancements—such as rituximab-based chemoimmunotherapy (e.g., R-CHOP) and novel targeted agents—have significantly improved OS, traditional tools such as the IPI remain insufficient for reliably predicting individual patient outcomes. These limitations highlight the urgent need for more precise and biologically informed prognostic models [18,19].

In this study, we systematically curated 1382 PCD-related genes from multiple sources including FerrDb V2, GSEA gene sets, and authoritative literature reviews. After rigorous univariate Cox regression and LASSO–Cox regression filtering, we identified 20 core prognostic PCD-related genes: *ARSG*, *BIRC3*, *CD70*, *CTH*, *ELL3*, *ERP29*, *FXN*, *HIF1A*, *INHBA*, *MAEL*, *MEF2C*, *MMP9*, *NLE1*, *PDK1*, *PDK4*, *PRKCA*, *RARRES2*, *RRAGA*, *SNAP23*, and *UBQLN2*. These genes are mechanistically diverse and participate in various aspects of cell fate decisions, metabolism, immune evasion, and tumor progression. For example, *ARSG*, *CTH*, *PDK1*, and *PDK4* are heavily involved in cellular metabolism and oxidative stress regulation, pathways tightly coupled with tumor proliferation and survival under adverse microenvironmental conditions [20,21,22,23,24,25]. Among them, *CTH* was further validated in our study as a functionally oncogenic gene; its knockdown significantly inhibited proliferation and induced apoptosis in DLBCL cells in vitro and suppressed tumor growth in vivo. These findings corroborate its role as a therapeutic target and mechanistic contributor to disease aggressiveness. Several other core genes also possess well-documented roles in apoptosis and immune modulation. For instance, *BIRC3*, *CD70*, *HIF1A*, *INHBA*, and *RARRES2* contribute to apoptosis regulation, immune modulation, and hypoxia responses [24,25,26,27,28]. Additionally, *ELL3*, *MAEL*, and *MEF2C* function as transcriptional regulators, influencing cellular development and gene expression [26,27,28]. Meanwhile, *MMP9* and *PRKCA* are implicated in extracellular matrix remodeling and metastasis [29,30], and *ERP29*, *FXN*, *SNAP23*, and *UBQLN2* maintain mitochondrial function and protein stability [31,32,33,34]. RRAGA, a key metabolic sensor, links metabolic activity to mTORC1 signaling and cell growth [35]. In addition, high expression of *ARSG*, *CD70*, *CTH*, *ERP29*, *FXN*, *NLE1*, and *PDK4* and low expression of *BIRC3*, *ELL3*, *HIF1A*, *INHBA*, *MAEL*, *MEF2C*, *MMP9*, *PDK1*, *PRKCA*, *RARRES2*, *RRAGA*, *SNAP23*, and *UBQLN2* are associated with poorer outcomes in DLBCL patients. Using these core genes, we established a novel prognostic score, the PCDS, and validated its reliability and independence, with the results providing insights into the prognostic and therapeutic landscape of DLBCL.

Although immunotherapy has revolutionized cancer treatment, its efficacy in DLBCL remains limited by the immunosuppressive TME and therapy resistance [36]. Our study highlighted the strong association between PCDS and the TME. Patients with a high PCDS exhibited significantly lower infiltration of anti-tumor immune cells, such as CD8+ T cells, dendritic cells, and M1 macrophages, and instead showed enrichment of immunosuppressive populations, particularly M2 macrophages. In addition, immune function analyses revealed diminished activity in critical pathways like APC co-stimulation and cytokine signaling, reinforcing the notion of a dysfunctional, “cold” immune milieu in high-PCDS tumors. These immunosuppressive characteristics were further supported by elevated expression of PD-1 and PD-L1, which positively correlated with the PCDS, implying an exhausted T cell state. Interestingly, survival analyses showed that patients with enriched M0 macrophages and γδ T cells had better outcomes, further underscoring the prognostic impact of the immune landscape [37,38,39].

Drug sensitivity analysis revealed PCDS-dependent heterogeneity in the predicted therapeutic responses. High-PCDS patients appeared more sensitive to 5-fluorouracil, fulvestrant, and sorafenib, whereas low-PCDS patients showed increased sensitivity to NU7441, a DNA-PK inhibitor. These findings suggest potential metabolic and DNA repair vulnerabilities associated with PCD-related dysregulation; however, they should be considered hypothesis-generating rather than definitive. Prospective validation in independent cohorts and experimental models will be required to confirm the clinical relevance of these drug susceptibility predictions.

Nonetheless, several limitations should be acknowledged. First, this study relied on retrospective datasets obtained from public databases, which may introduce selection bias and limit generalizability. Second, although *CTH* was experimentally validated, additional in vitro and in vivo functional studies are needed to elucidate the biological roles of the other core genes. Third, the model’s clinical utility needs validation in multi-center, prospective cohorts to confirm its predictive accuracy across different populations and treatment regimens. Moreover, integrating PCDSs with additional biomarkers (e.g., circulating tumor DNA, immune signatures) may further refine its prognostic value.

## 5. Conclusions

Our study provides a comprehensive analysis of PCD signatures in DLBCL and establishes a novel prognostic model—the PCDS. This score functions as an independent prognostic biomarker and reflects the underlying immunological and pharmacological heterogeneity of the disease. A high PCDS is associated with immune suppression, altered drug sensitivity, and unfavorable clinical outcomes, offering mechanistic insights into disease biology. While these findings highlight the potential value of incorporating PCD signatures into future stratification frameworks, further validation in prospective and multi-center studies will be essential before translation into routine clinical practice.

## Data Availability

The data presented in this study are available in the GEO database at https://www.ncbi.nlm.nih.gov/geo/, reference numbers GSE10846, GSE11318, and GSE87371 (accessed on 1 June 2025). These data were derived from publicly available resources in the GEO repository.

## Supplementary Materials

The following supporting information can be downloaded at https://www.mdpi.com/article/10.3390/biomedicines13102320/s1, Table S1: A comprehensive list of PCD-related genes in DLBCL. Table S2: Prognosis-associated genes identified in DLBCL by univariate Cox regression analysis. Figure S1: Distribution of PCDS across clinical subgroups of DLBCL patients. Figure S2: Pathway enrichment analysis between high- and low-PCDS groups. Figure S3: Kaplan–Meier survival analysis across clinical subgroups in multiple DLBCL cohorts. Figure S4: Prognostic impact of immune cell infiltration in DLBCL patients.

## Abbreviations

The following abbreviations are used in this manuscript:

ABC	Activated B cell-like
ATCC	American Type Culture Collection
Annexin V-FITC	Annexin V conjugated with fluorescein isothiocyanate
AUC	Area under the curve
aDCs	Activated dendritic cell
APC	Antigen-presenting cell
CI	Confidence interval
C-index	Concordance index
CCR	Cytokine–cytokine receptor interaction
DLBCL	Diffuse large B cell lymphoma
DAMPs	Damage-associated molecular patterns
ECOG	Eastern Cooperative Oncology Group
EdU	5-Ethynyl-2′-deoxyuridine
GCB	Germinal center B cell-like
GSCA	Gene Set Cancer Analysis
GSEA	Gene set enrichment analysis
HRs	Hazard ratios
IC_50_	Half maximal inhibitory concentration
IFN	Interferon
LDH	Lactate dehydrogenase
IPI	International Prognostic Index
OS	Overall survival
PCD	Programmed cell death
PCDS	Programmed cell death score
PCA	Principal component analysis
PD-1	Programmed cell death protein 1
PD-L1	Programmed cell death ligand 1
PI	Propidium iodide
ROC	Receiver operating characteristic
STR	Short tandem repeat
TME	Tumor microenvironment

## References

[1] Shi Y., Xu Y., Shen H., Jin J., Tong H., Xie W. (2024). Advances in biology, diagnosis and treatment of DLBCL. Ann. Hematol..

[2] Xu P.P., Huo Y.J., Zhao W.L. (2022). All roads lead to targeted diffuse large B-cell lymphoma approaches. Cancer Cell.

[3] Tang D., Kang R., Berghe T.V., Vandenabeele P., Kroemer G. (2019). The molecular machinery of regulated cell death. Cell Res..

[4] Liu X., Nie L., Zhang Y., Yan Y., Wang C., Colic M., Olszewski K., Horbath A., Chen X., Lei G. (2023). Actin cytoskeleton vulnerability to disulfide stress mediates disulfidptosis. Nat. Cell Biol..

[5] Xu X., Lai Y., Hua Z.C. (2019). Apoptosis and apoptotic body: Disease message and therapeutic target potentials. Biosci. Rep..

[6] D’Arcy M.S. (2019). Cell death: A review of the major forms of apoptosis, necrosis and autophagy. Cell Biol. Int..

[7] Liang Y., Zhao Y., Qi Z., Li X., Zhao Y. (2025). Ferroptosis: CD8(+)T cells’ blade to destroy tumor cells or poison for self-destruction. Cell Death Discov..

[8] Hu Y., Yu Q., Li X., Wang J., Guo L., Huang L., Gao W. (2025). Nanoformula Design for Inducing Non-Apoptotic Cell Death Regulation: A Powerful Booster for Cancer Immunotherapy. Adv. Healthc. Mater..

[9] Wang S., Guo S., Guo J., Du Q., Wu C., Wu Y., Zhang Y. (2024). Cell death pathways: Molecular mechanisms and therapeutic targets for cancer. MedComm (2020).

[10] Hu Y., Liu Y., Zong L., Zhang W., Liu R., Xing Q., Liu Z., Yan Q., Li W., Lei H. (2023). The multifaceted roles of GSDME-mediated pyroptosis in cancer: Therapeutic strategies and persisting obstacles. Cell Death Dis..

[11] Zheng N., Fang J., Xue G., Wang Z., Li X., Zhou M., Jin G., Rahman M.M., McFadden G., Lu Y. (2022). Induction of tumor cell autosis by myxoma virus-infected CAR-T and TCR-T cells to overcome primary and acquired resistance. Cancer Cell.

[12] Kahlson M.A., Dixon S.J. (2022). Copper-induced cell death. Science.

[13] Jing Z., Huang W., Mei J., Bhushan S., Wu X., Yan C., Zheng H., Yang Y. (2025). Advances in novel cell death mechanisms in breast cancer: Intersecting perspectives on ferroptosis, cuproptosis, disulfidptosis, and pyroptosis. Mol. Cancer.

[14] Jaillon S., Ponzetta A., Di Mitri D., Santoni A., Bonecchi R., Mantovani A. (2020). Neutrophil diversity and plasticity in tumour progression and therapy. Nat. Rev. Cancer.

[15] Huntington N.D., Cursons J., Rautela J. (2020). The cancer-natural killer cell immunity cycle. Nat. Rev. Cancer.

[16] DeNardo D.G., Ruffell B. (2019). Macrophages as regulators of tumour immunity and immunotherapy. Nat. Rev. Immunol..

[17] Jhunjhunwala S., Hammer C., Delamarre L. (2021). Antigen presentation in cancer: Insights into tumour immunogenicity and immune evasion. Nat. Rev. Cancer.

[18] Wight J.C., Chong G., Grigg A.P., Hawkes E.A. (2018). Prognostication of diffuse large B-cell lymphoma in the molecular era: Moving beyond the IPI. Blood Rev..

[19] Wright G.W., Huang D.W., Phelan J.D., Coulibaly Z.A., Roulland S., Young R.M., Wang J.Q., Schmitz R., Morin R.D., Tang J. (2020). A Probabilistic Classification Tool for Genetic Subtypes of Diffuse Large B Cell Lymphoma with Therapeutic Implications. Cancer Cell.

[20] Kowalewski B., Lübke T., Kollmann K., Braulke T., Reinheckel T., Dierks T., Damme M. (2014). Molecular characterization of arylsulfatase G: Expression, processing, glycosylation, transport, and activity. J. Biol. Chem..

[21] Peleli M., Antoniadou I., Rodrigues-Junior D.M., Savvoulidou O., Caja L., Katsouda A., Ketelhuth D.F.J., Stubbe J., Madsen K., Moustakas A. (2023). Cystathionine gamma-lyase (*CTH*) inhibition attenuates glioblastoma formation. Redox Biol..

[22] Kim J.W., Tchernyshyov I., Semenza G.L., Dang C.V. (2006). HIF-1-mediated expression of pyruvate dehydrogenase kinase: A metabolic switch required for cellular adaptation to hypoxia. Cell Metab..

[23] Jiang Q., Zheng N., Bu L., Zhang X., Zhang X., Wu Y., Su Y., Wang L., Zhang X., Ren S. (2021). SPOP-mediated ubiquitination and degradation of PDK1 suppresses AKT kinase activity and oncogenic functions. Mol. Cancer.

[24] Dou X., Fu Q., Long Q., Liu S., Zou Y., Fu D., Xu Q., Jiang Z., Ren X., Zhang G. (2023). PDK4-dependent hypercatabolism and lactate production of senescent cells promotes cancer malignancy. Nat. Metab..

[25] Ma W.Q., Sun X.J., Zhu Y., Liu N.F. (2020). PDK4 promotes vascular calcification by interfering with autophagic activity and metabolic reprogramming. Cell Death Dis..

[26] Alexander L.M.M., Watters J., Reusch J.A., Maurin M., Nepon-Sixt B.S., Vrzalikova K., Alexandrow M.G., Murray P.G., Wright K.L. (2017). Selective expression of the transcription elongation factor ELL3 in B cells prior to ELL2 drives proliferation and survival. Mol. Immunol..

[27] Zhou L., Ou S., Liang T., Li M., Xiao P., Cheng J., Zhou J., Yuan L. (2023). MAEL facilitates metabolic reprogramming and breast cancer progression by promoting the degradation of citrate synthase and fumarate hydratase via chaperone-mediated autophagy. FEBS J..

[28] Bao Z., Hua L., Ye Y., Wang D., Li C., Xie Q., Wakimoto H., Gong Y., Ji J. (2021). MEF2C silencing downregulates NF2 and E-cadherin and enhances Erastin-induced ferroptosis in meningioma. Neuro Oncol..

[29] Huang H. (2018). Matrix Metalloproteinase-9 (MMP-9) as a Cancer Biomarker and MMP-9 Biosensors: Recent Advances. Sensors.

[30] Li J.X., Li R.Z., Sun A., Zhou H., Neher E., Yang J.S., Huang J.M., Zhang Y.Z., Jiang Z.B., Liang T.L. (2021). Metabolomics and integrated network pharmacology analysis reveal Tricin as the active anti-cancer component of Weijing decoction by suppression of PRKCA and sphingolipid signaling. Pharmacol. Res..

[31] Zhang D., Richardson D.R. (2011). Endoplasmic reticulum protein 29 (ERp29): An emerging role in cancer. Int. J. Biochem. Cell Biol..

[32] Du J., Zhou Y., Li Y., Xia J., Chen Y., Chen S., Wang X., Sun W., Wang T., Ren X. (2020). Identification of Frataxin as a regulator of ferroptosis. Redox Biol..

[33] Dolai S., Takahashi T., Qin T., Liang T., Xie L., Kang F., Miao Y.F., Xie H., Kang Y., Manuel J. (2021). Pancreas-specific SNAP23 depletion prevents pancreatitis by attenuating pathological basolateral exocytosis and formation of trypsin-activating autolysosomes. Autophagy.

[34] Shah P.P., Saurabh K., Kurlawala Z., Vega A.A., Siskind L.J., Beverly L.J. (2022). Towards a molecular understanding of the overlapping and distinct roles of UBQLN1 and UBQLN2 in lung cancer progression and metastasis. Neoplasia.

[35] Chen J.H., Huang C., Zhang B., Yin S., Liang J., Xu C., Huang Y., Cen L.P., Ng T.K., Zheng C. (2016). Mutations of RagA GTPase in mTORC1 Pathway Are Associated with Autosomal Dominant Cataracts. PLoS Genet..

[36] Zhang Y., Zhang Z. (2020). The history and advances in cancer immunotherapy: Understanding the characteristics of tumor-infiltrating immune cells and their therapeutic implications. Cell Mol. Immunol..

[37] Wang T.W., Johmura Y., Suzuki N., Omori S., Migita T., Yamaguchi K., Hatakeyama S., Yamazaki S., Shimizu E., Imoto S. (2022). Blocking PD-L1-PD-1 improves senescence surveillance and ageing phenotypes. Nature.

[38] Cha J.H., Chan L.C., Li C.W., Hsu J.L., Hung M.C. (2019). Mechanisms Controlling PD-L1 Expression in Cancer. Mol. Cell.

[39] Jiang X., Wang J., Deng X., Xiong F., Ge J., Xiang B., Wu X., Ma J., Zhou M., Li X. (2019). Role of the tumor microenvironment in PD-L1/PD-1-mediated tumor immune escape. Mol. Cancer.

