# Supplementary material for: Programmed-Cell-Death-Related Signature Reveals Immune Microenvironment Characteristics and Predicts Therapeutic Response in Diffuse Large B Cell Lymphoma
Source: Biomedicines. 2025 Sep 23;13(10):2320. doi: 10.3390/biomedicines13102320 (PMC12562125; doi:10.3390/biomedicines13102320)
Supplement: Supplementary file 1 [file biomedicines-13-02320-s001.zip › Supplementary Figure legends.pdf]

Supplementary Material

Table S1.

A comprehensive list of PCD-related genes in DLBCL.

Table S2.

Prognosis-associated genes identified in DLBCL by univariate Cox regression analysis.

Figure S1. Distribution of PCDS across clinical subgroups of DLBCL patients.

(A-B) Distribution of PCDS among DLBCL patients stratified by clinical factors, including age (< 60 vs. ≥ 60 years) and disease stage (I–II vs. III–IV). ns, not significant;. * $p < 0.05$; ** $p < 0.01$; *** $p < 0.001$.

Figure S2. Pathway enrichment analysis between high- and low-PCDS groups.

(A-B) GSEA showing the top five enriched pathways in the GSE10846 dataset, comparing high- and low-PCDS groups.

Figure S3. Kaplan–Meier survival analysis across clinical subgroups in multiple DLBCL cohorts.

(A-C) Kaplan–Meier curves showing OS of DLBCL patients in three independent cohorts: GSE10846 (n = 412), GSE11318 (n = 199), and GSE87371 (n = 221). Patients were stratified into high- and low-PCDS groups within clinical subgroups, including age (> 60 vs. ≤ 60 years) and Ann Arbor stage (I–II vs. III–IV).

Figure S4. Prognostic impact of immune cell infiltration in DLBCL patients.

(A-C) Kaplan-Meier survival curves stratified by infiltration levels of (A) M0 macrophages, (B) T cells gamma delta , and (C) M2 macrophages in DLBCL patients.
