# Supplementary figures and images for: Programmed-Cell-Death-Related Signature Reveals Immune Microenvironment Characteristics and Predicts Therapeutic Response in Diffuse Large B Cell Lymphoma
Source: Biomedicines. 2025 Sep 23;13(10):2320. doi: 10.3390/biomedicines13102320 (PMC12562125; doi:10.3390/biomedicines13102320)

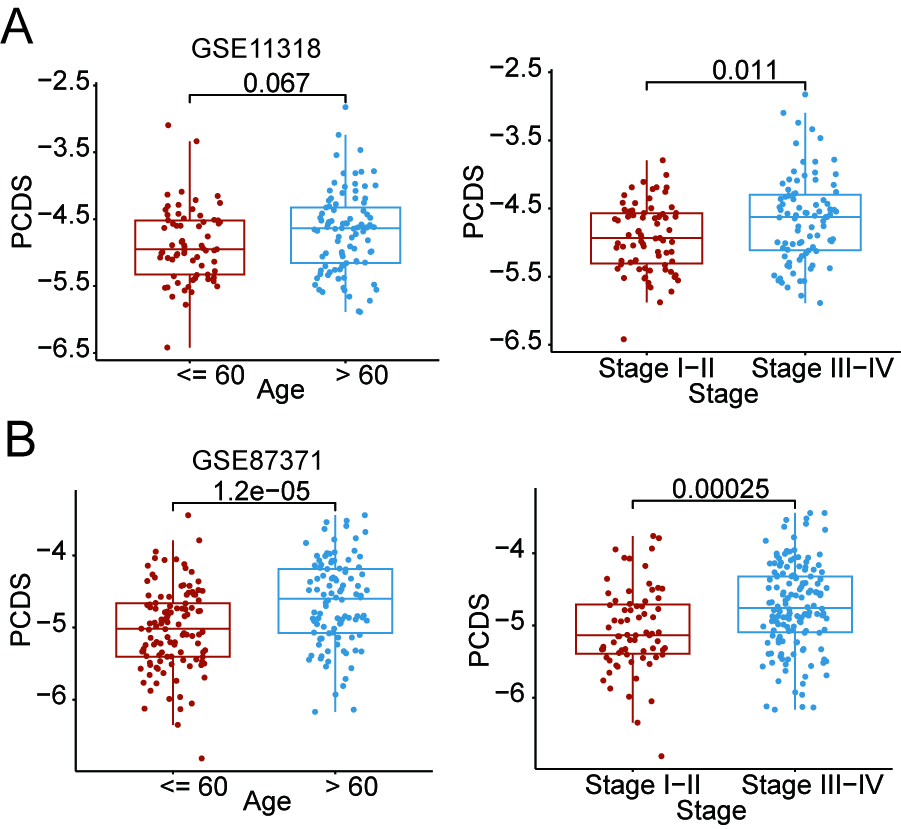

Supplement: Supplementary file 1 [file biomedicines-13-02320-s001.zip › Figure S1.tif]

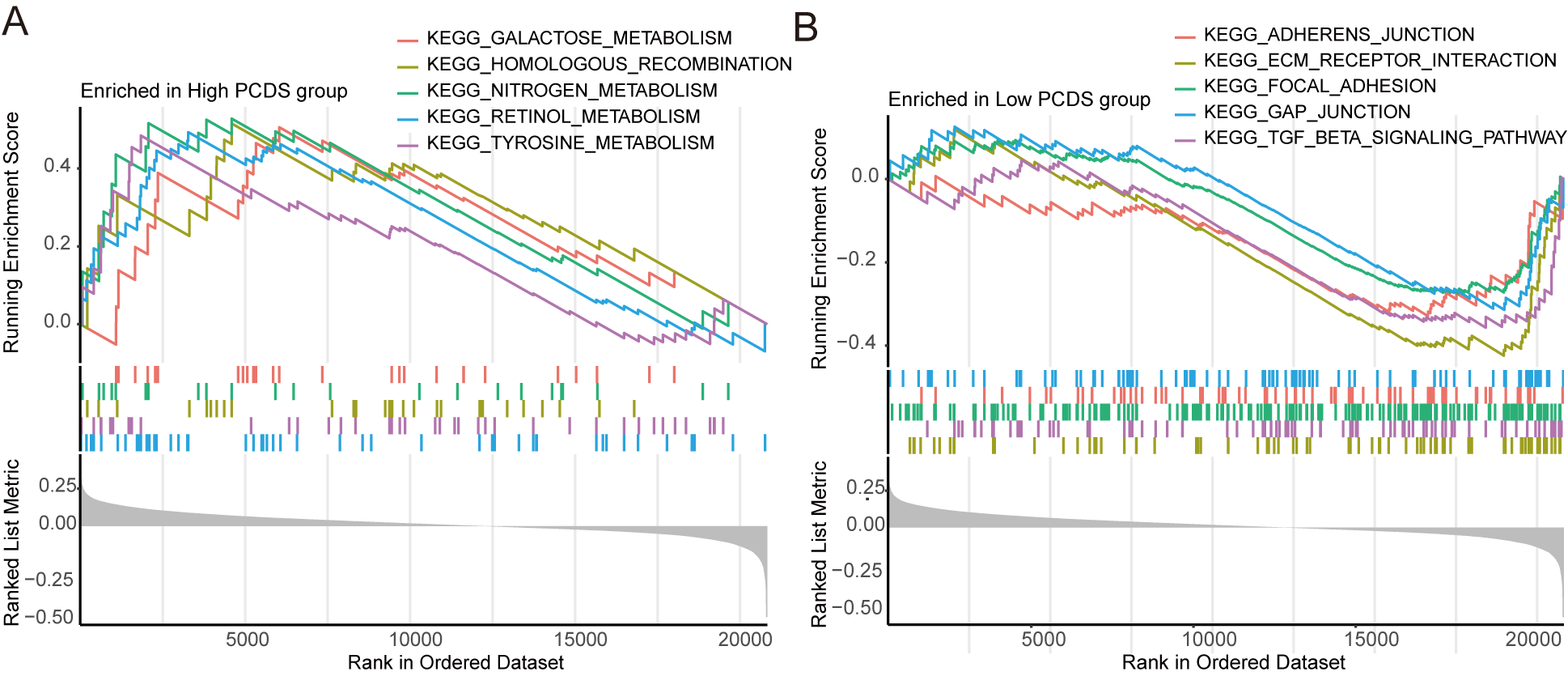

Supplement: Supplementary file 1 [file biomedicines-13-02320-s001.zip › Figure S2.tif]

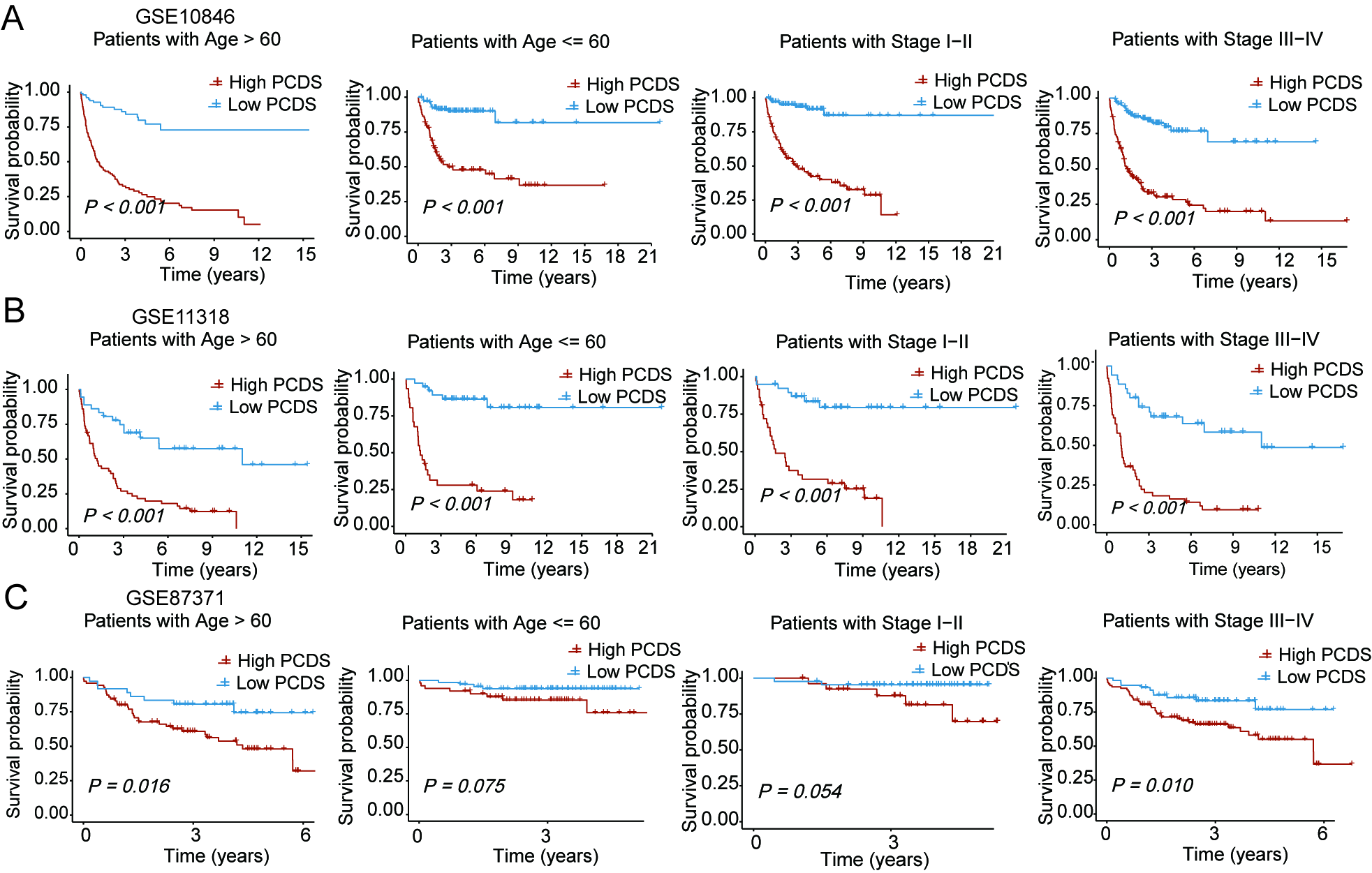

Supplement: Supplementary file 1 [file biomedicines-13-02320-s001.zip › Figure S3.tif]

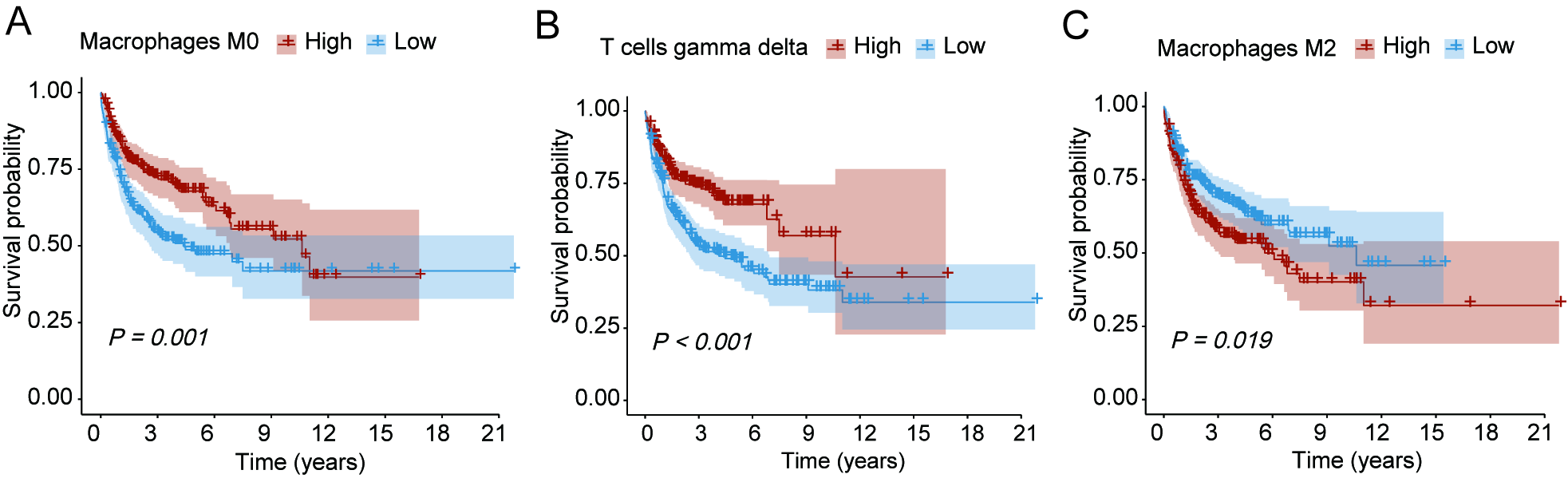

Supplement: Supplementary file 1 [file biomedicines-13-02320-s001.zip › Figure S4.tif]
